# Supplementary material for: Genome-wide identification, characterization and gene expression of BES1 transcription factor family in grapevine (Vitis vinifera L.)
Source: Sci Rep. 2023 Jan 5;13:240. doi: 10.1038/s41598-022-24407-y (PMC9816167; doi:10.1038/s41598-022-24407-y)
Supplement: Supplementary file 3 — Supplementary Information. [file 41598_2022_24407_MOESM3_ESM.zip › Vvi_Atr/Vitis_vinifera.PN40024.v4.dna_sm.toplevel.fa.vs.Amborella_trichopoda.AMTR1.0.dna_sm.toplevel.fa.html/Atr-AmTr_v1.0_scaffold00164.html]

|  |  |  |  |  |  |  |  |  |  |  |  |  |  |
| --- | --- | --- | --- | --- | --- | --- | --- | --- | --- | --- | --- | --- | --- |
| Duplication depth | Reference chromosome | Collinear blocks | | | | | | | | | | | |
| 0 | Atr-ERN10898 |  |  |  |  |  |  |  |  |  |  |  |  |
| 1 | Atr-ERN10899 |  | Vvi-Vitvi18g00667\_t001 |  |  |  |  |  |  |  |  |  |  |
| 1 | Atr-ERN10900 |  | \| \| |  |  |  |  |  |  |  |  |  |  |
| 1 | Atr-ERN10901 |  | \| \| |  |  |  |  |  |  |  |  |  |  |
| 1 | Atr-ERN10902 |  | Vvi-Vitvi18g00668\_t003 |  |  |  |  |  |  |  |  |  |  |
| 1 | Atr-ERN10903 |  | \| \| |  |  |  |  |  |  |  |  |  |  |
| 1 | Atr-ERN10904 |  | \| \| |  |  |  |  |  |  |  |  |  |  |
| 2 | Atr-ERN10905 |  | Vvi-Vitvi18g02649\_t001 |  | Vvi-Vitvi03g00675\_t001 |  |  |  |  |  |  |  |  |
| 2 | Atr-ERN10906 |  | \| \| |  | \| \| |  |  |  |  |  |  |  |  |
| 2 | Atr-ERN10907 |  | Vvi-Vitvi18g00670\_t001 |  | Vvi-Vitvi03g00639\_t001 |  |  |  |  |  |  |  |  |
| 2 | Atr-ERN10908 |  | \| \| |  | \| \| |  |  |  |  |  |  |  |  |
| 2 | Atr-ERN10909 |  | \| \| |  | \| \| |  |  |  |  |  |  |  |  |
| 2 | Atr-ERN10910 |  | \| \| |  | \| \| |  |  |  |  |  |  |  |  |
| 2 | Atr-ERN10911 |  | \| \| |  | \| \| |  |  |  |  |  |  |  |  |
| 2 | Atr-ERN10912 |  | \| \| |  | \| \| |  |  |  |  |  |  |  |  |
| 2 | Atr-ERN10913 |  | \| \| |  | \| \| |  |  |  |  |  |  |  |  |
| 2 | Atr-ERN10914 |  | \| \| |  | \| \| |  |  |  |  |  |  |  |  |
| 2 | Atr-ERN10915 |  | Vvi-Vitvi18g00671\_t001 |  | \| \| |  |  |  |  |  |  |  |  |
| 2 | Atr-ERN10916 |  | \| \| |  | \| \| |  |  |  |  |  |  |  |  |
| 2 | Atr-ERN10917 |  | \| \| |  | \| \| |  |  |  |  |  |  |  |  |
| 2 | Atr-ERN10918 |  | \| \| |  | \| \| |  |  |  |  |  |  |  |  |
| 2 | Atr-ERN10919 |  | \| \| |  | \| \| |  |  |  |  |  |  |  |  |
| 2 | Atr-ERN10920 |  | \| \| |  | \| \| |  |  |  |  |  |  |  |  |
| 2 | Atr-ERN10921 |  | \| \| |  | \| \| |  |  |  |  |  |  |  |  |
| 2 | Atr-ERN10922 |  | \| \| |  | Vvi-Vitvi03g00636\_t001 |  |  |  |  |  |  |  |  |
| 2 | Atr-ERN10923 |  | \| \| |  | \| \| |  |  |  |  |  |  |  |  |
| 2 | Atr-ERN10924 |  | Vvi-Vitvi18g00673\_t001 |  | Vvi-Vitvi03g00635\_t001 |  |  |  |  |  |  |  |  |
| 1 | Atr-ERN10925 |  |  |  | Vvi-Vitvi03g00634\_t001 |  |  |  |  |  |  |  |  |
| 1 | Atr-ERN10926 |  |  |  | Vvi-Vitvi03g00633\_t001 |  |  |  |  |  |  |  |  |
| 1 | Atr-ERN10927 |  |  |  | Vvi-Vitvi03g00631\_t001 |  |  |  |  |  |  |  |  |
| 0 | Atr-ERN10928 |  |  |  |  |  |  |  |  |  |  |  |  |
| 0 | Atr-ERN10929 |  |  |  |  |  |  |  |  |  |  |  |  |
| 0 | Atr-ERN10930 |  |  |  |  |  |  |  |  |  |  |  |  |
| 0 | Atr-ERN10931 |  |  |  |  |  |  |  |  |  |  |  |  |
| 0 | Atr-ERN10932 |  |  |  |  |  |  |  |  |  |  |  |  |
| 0 | Atr-ERN10933 |  |  |  |  |  |  |  |  |  |  |  |  |
| 0 | Atr-ERN10934 |  |  |  |  |  |  |  |  |  |  |  |  |
| 0 | Atr-ERN10935 |  |  |  |  |  |  |  |  |  |  |  |  |
